# Editorial Note

**Published:** 1947

**Authors:** 


### Editorial Note

**The Future of the "Journal."**

The future of this *Journal* has been discussed at two meetings of the Society and at several meetings of its Committee. Two main questions were faced: one being whether the *Journal* was of value to the Society and the Medical Profession in the neighbourhood; the second being the question of cost of production.

The Committee came to the unanimous opinion that the *Journal* should continue to be published quarterly as heretofore and made a recommendation that enquires should be instituted to ascertain whether the *Journal* might become again what it was at its foundation : a journal for the South West of England as well as for Bristol.

The Society at a well-attended meeting on November 12th agreed to the Committee's recommendation. In order to meet rising costs, the Anual Subscription to the Society was increased from one guinea to a guinea and a half. This is the figure to which it was increased after the 1914-18 war, until 1936.

The financial position causes no anxiety. During the eight years 1939-47 the average cost of each year was £183 10s. and it is estimated that in future four quarterly numbers can be produced for about £300. For comparision it is interesting to look back to the year 1898 and find that £206 17s. 10d. was spent on the *Journal.* The relatively low cost during the last war years was due to a smaller number of issues and the scanty material at the disposal of the Editorial Committee.

The *Bristol Medico-Chirurgical Journal* is completing its sixty-fourth year of publication and not even two World Wars have interrupted its appearance, even though its regularity has not been fully maintained.

This journal has always endeavoured to publish matter of interest to general practitioners as well as to specialists.

The Society and the Editorial Committee will welcome ideas and suggestions on this matter.

Any reader, whether regular or occasional, who has ideas to contribute should write to the Editor. The correspondence will not be published owing to limitations of space and paper, but any letters will receive grateful and careful consideration.

